# A Survey of Internet of Things (IoT) Authentication Schemes †

**DOI:** 10.3390/s19051141

**Published:** 2019-03-06

**Authors:** Mohammed El-hajj, Ahmad Fadlallah, Maroun Chamoun, Ahmed Serhrouchni

**Affiliations:** 1 University of Sciences and Arts in Lebanon, Beirut 1002, Lebanon; a.fadlallah@usal.edu.lb; 2 Saint Joseph University, Beirut 1514, Lebanon; maroun.chamoun@usj.edu.lb; 3 Telecom ParisTech, 75013 Paris, France

**Keywords:** Internet of Things, IoT, security, authentication

## Abstract

The Internet of Things (IoT) is the ability to provide everyday devices with a way of identification and another way for communication with each other. The spectrum of IoT application domains is very large including smart homes, smart cities, wearables, e-health, etc. Consequently, tens and even hundreds of billions of devices will be connected. Such devices will have smart capabilities to collect, analyze and even make decisions without any human interaction. Security is a supreme requirement in such circumstances, and in particular authentication is of high interest given the damage that could happen from a malicious unauthenticated device in an IoT system. This paper gives a near complete and up-to-date view of the IoT authentication field. It provides a summary of a large range of authentication protocols proposed in the literature. Using a multi-criteria classification previously introduced in our work, it compares and evaluates the proposed authentication protocols, showing their strengths and weaknesses, which constitutes a fundamental first step for researchers and developers addressing this domain.

## 1. Introduction

The number of connected devices is growing exponentially, forming the so-called Internet of Things (IoT), a large network of networks connecting smart devices such as sensors and actuators. Such devices are adopted in various domains such as public health, smart grids, smart transportation, waste management, smart homes, smart cities, agriculture, energy management, etc. [1,2,3].

The requirements and limitations of the connected “things” raise a number of challenges, including *connectivity challenges* for billions of devices to communicate with each other, *security challenges* with the need to protect IoT networks from being attacked (according to a Gartner report, 20% of organizations have experienced at least one IoT attack in the last three years [4]) and at the same time from being exploited to become an attack tool (e.g., Mirai botnet [5,6]). These challenges are “augmented” with the resource-limited nature of IoT devices which renders traditional communication protocols and security schemes inefficient and even infeasible for IoT. The IoT-related security issues are becoming more alarming given the ubiquitousness of IoT devices and their adoption in critical applications, which aggravate the impact of any security breach to the extent of being life-threatening. A sample scenario can be foreseen from a weakness discovered in 2017 in a pacemaker device, which led to the recall done by US Food and Drug Administration (FDA) of 500,000 pacemakers having a dread that security gaps could cause a hacker to control the heart-beat regulating device [7].

The security requirements of an IoT network mainly depend on the type of applications it serves; the need for confidentiality, integrity and/or authentication directly depends on the security needs of the application. In particular, authentication is considered as a key requirement for IoT [3]; trusting the devices participating in an IoT network is crucial for the well-functioning of the network. A single compromised node can be turned malicious and bring down the whole system or cause disasters [1].

The specific nature of IoT devices makes the traditional authentication schemes infeasible and not applicable. Indeed, cryptographic schemes designed for main-powered, high processing and/or large memory devices do not “suit” the resource-limited IoT nodes. This led to the emergence of lightweight authentication schemes; some of them are specific to the context of IoT or to the Wireless Sensor Network (WSN) (which can be considered suitable for IoT).

This paper presents a general overview of the security concerns and requirements in the IoT environment in a layer-based approach. Then, it provides an up-to-date survey of the different IoT authentication schemes. Through a multi-criteria classification, it compares and analyzes the existing authentication protocols, showing their advantages and disadvantages, which is an extension of a previously published research work [1,2].

The rest of this paper is organized as follow: The generic architecture of IoT is presented in Section 2. Section 3 discusses the main security concerns in IoT and the security challenges at each layer of the IoT architecture. Section 4 provides a taxonomy of the existing authentication schemes, while Section 5 analyzes the most-known IoT authentication schemes in light of the proposed taxonomy. Section 6 provides an overview of the related works. Finally, Section 7 concludes the paper and discusses the findings of the survey.

## 2. IoT Generic Architecture

While traditional Internet connects people to a network, IoT has a different approach in which it provides Machine-to-Machine (M2M) and Human-to-Machine (H2M) connectivity, for heterogeneous types of machines in order to support variety of applications (e.g., identifying, locating, tracking, monitoring, and controlling) [8]. Connecting a huge number of heterogeneous machines [9] leads to a massive traffic, hence the need to deal with the storage of big data [10,11]. Therefore, the TCP/IP architecture, which has been used for a long time for network connectivity, does not suit the needs of IoT regarding various aspects including privacy and security (e.g., information privacy, machine’s safety, data confidentiality, data encryption, and network security) [12], scalability, reliability, interoperability, and quality of service [13].

**Figure 1:**
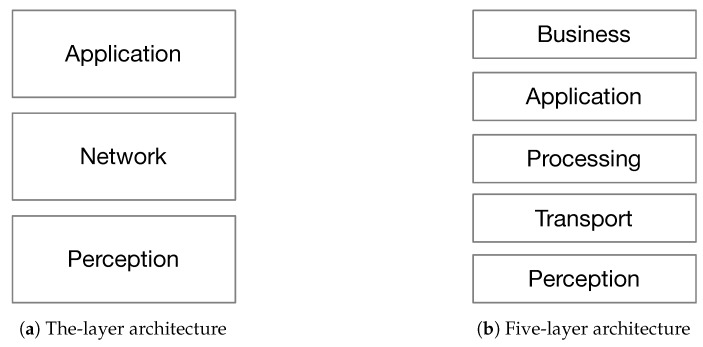
IoT architecture models.

Although numerous architectures were proposed for IoT, there is still a need for a reference architecture [14,15]. The basic architecture model proposed in the literature is a three-layer architecture [13,16,17,18], as shown in Figure 1a. It consists of: perception, network and application layers.

1. *Perception layer*: It is the physical layer that senses the environment to perceive the physical properties (e.g., temperature, humidity, speed, location, etc.) using end-nodes, through the use of different sensing technologies (e.g., RFID, GPS, NFC, etc.).
2. *Network Layer*: It is the layer in charge of getting data from the perception layer and transmitting it to the application layer through various network technologies (e.g., 3G, 4G, 5G, Wi-Fi, Bluetooth, Zig-Bee, etc.). It is also responsible of data management from storing to processing with the help of middle-wares such as cloud computing.
3. *Application Layer*: It is the layer that is in charge of delivering application-specific services to the user. The importance of this layer is that it has the ability to cover numerous markets (e.g., smart cities, smart homes, health care, building automation, smart metering, etc. [1,2]) [19].

Another proposed layered architecture is the five-layer architecture (Figure 1b) [13,16,17,18]. The five layers are from top to bottom: *business*, *application*, *processing*, *transport*, and *perception* layers. The functions of perception, transport (i.e., network layer) and application layers are the same as in the three-layer architecture. The remaining layers of the architecture are:

1. *Processing layer*: Also called the middle-ware layer, it is responsible of providing various types of services, mainly storing, analyzing, and processing data with respect to the computational results.
2. *Business layer*: Its work covers the overall IoT system actions and functionality. The application layer sends the data to the business layer whose role is to build business models, graphs, and flowcharts to analyze data, in order to play a role in decision making about business strategies and road-maps.

Other architectures can also be identified in the literature. In [20,21], the authors used a five-layer architecture based on Service Oriented Architecture (SOA) that helps the integration of IoT in enterprise services. In [22], the authors considered a non-layered approach for the architecture (e.g., cloud architecture, fog architecture, social IoT, and architecture based on human brain processing).

For the rest of this paper, we consider the three-layer architecture.

## 3. Security Issues in IoT

### 3.1. Security Services

As previously mentioned, the use of connecting objects in everyday people’s lives can make security issues life-threatening. The smartness integrated into homes, cars, and electric grids can be diverted into harmful scenarios when exploited by hackers. Different hacking scenarios presented in the past years [23,24] illustrate the level of harm that could result from a security breach, especially with the development and large adoption of IoT applications dealing with sensitive information (personal, industrial, governmental, etc.).

The main IoT security concerns are: authentication, authorization, integrity, confidentiality, non-repudiation, Availability, and privacy [25,26,27].

1. *Authentication*: The process of confirming and insuring the identity of objects. In IoT context, each object should have the ability to identify and authenticate all other objects in the system (or in a given part of the system with which it interacts).
2. *The authorization*: The process of giving permission to an entity to do or have something [28].
3. *Integrity*: The way toward keeping up the consistency, precision and dependability of information over its whole life cycle. In IoT, the alteration of basic information or even the infusion of invalid information could prompt major issues, e.g., in smart health systems use cases it could lead to the death of the patient [29].
4. *Confidentiality*: The process of ensuring that the information is only accessed by authorized people. Two main issues should be considered [27] regarding confidentiality in IoT: firstly, to ensure that the object receiving the data is not going to move/transfer these data to other objects and, secondly, to consider the data management.
5. *Non-repudiation*: The way toward guaranteeing the ability to demonstrate that a task or event has occurred (and by whom), with the goal that this cannot be denied later. In other words, the object cannot deny the authenticity of a specific data transferred.
6. *Availability*: The process of ensuring that the service needed is available anywhere and anytime for the intended users. This includes in IoT, the availability of the objects themselves.
7. *Privacy*: The process of ensuring non-accessibility to private information by public or malicious objects [30].

### 3.2. Security Challenges in IoT Layers

In this section, we consider the most basic architecture of IoT (three-layer architecture), and discuss the security concerns, attacks and security requirements at each layer of the architecture.

#### 3.2.1. Perception Layer Security Issues and Requirements

The perception layer consists of sensors that are characterized by limited processing power and storage capacity [31]. Several security issues and attack risks rise due to such limitations.

Several attacks on the perception layer are noticed:

1. *Node Capture*: Nodes (base node or gateway) can be easily controlled by the attackers. Catching a node empowers an adversary not only to get tightly of cryptographic keys and protocol states, but also to clone and redistribute malicious nodes in the network, which affects the security of the entire network [32,33].
2. *Denial of Service (DoS) Attack*: A type of attacks that shuts down the system or network and prevents authorized users from accessing it. This could be achieved by overwhelming the system or network with large amount of spam requests all at the same time, thus overloading the system and preventing it from delivering the normal service [34].
3. *Denial of Sleep Attack*: One of the essential objective of an IoT network is the capability of sensing through an extensive number of distributed nodes, each providing small data, such as temperature, humidity, vibration, etc. at a set interval and then going to sleep for another time interval in order to allow the nodes to operate for long service life. The denial of sleep attack works on the power supply of the node with a significant goal to increase the power consumption in order to reduce the service lifetime of the node by preventing the node from going asleep after sending the appropriate sensed data [35,36].
4. *Distributed Denial of Service (DDoS) Attack*: A large scale variant of DoS attacks. The most challenging issue is the ability to use the large amount of IoT nodes to pass traffic collected toward the victim server [37,38]. There are indications that the DDoS attack called “Mirai” [5,6] occuring on October 2016 benefited from a large number of IoT nodes.
5. *Fake Node/Sybil Attack*: A type of attacks where the attacker can deploy fake identities using fake nodes. With the presence of a sybil node, the whole system might generate wrong data or even the neighbor nodes will receive spam data and will mislay their privacy [39,40]. The fake nodes could be used to transmit data to “legitimate” nodes leading them to consume their energy, which could lead the whole service to go down.
6. *Replay Attack*: In this attack, information is stored and re-transmitted later without having the authority to do that. Such attacks are commonly used against authentication protocols [41,42].
7. *Routing Threats*: This type of attacks is the most fundamental attack at the network layer but it could occur at the perception layer in data forwarding process. An attacker can create a routing loop causing the shortage or extension of the routing path, increasing the end-to-end delay, and increasing the error messages [43].
8. *Side-Channel Attack*: This type of attacks occurs on encryption devices by taking advantage of the hardware information where the crypto-system is applied on (chips), such as the execution time, power consumption, power dissipation, and electromagnetic interference produced by electronic devices throughout the encryption procedure. Such information could be analyzed to discover secret keys used during the encryption process [44,45,46,47].
9. *Mass Node Authentication*: The process of authenticating large amount of devices in an IoT system, which requires massive amount of network communication for the authentication phase to finish and this could affect the performance of the whole system.

Taking into consideration the above-mentioned risks, there is a need for node authentication to prevent fake node and illegal access, in addition to the need for data encryption to protect the confidentiality of data while being transmitted between nodes (end node, gateway or server). Due to the properties of the nodes with respect to the shortage of power and the limited storage capacity, there is a necessity for mature lightweight security schemes that include both lightweight cryptographic algorithms and security protocols.

#### 3.2.2. Network Layer Security Issues and Requirements

The network layer is in charge of the diffusion of data from the perception layer to the application layer. This is where data routing occurs as well as the primary data analysis. In this layer, several network technologies are used such as the different technologies for mobile communication generations (2G, 3G, 4G and 5G) and wireless networks (Bluetooth, WiMAX, WiFi, LoRaWAN, etc.).

Several attacks and risks on the network layer are identified:

1. *Man-in-the-Middle (MITM)*: According to McAfee [6], the most recurrent attacks are Denial of Service (DoS) and Man In the Browser (MITB) attacks. This latter, along with the Secure Socket Layer (SSL) attack, which enables attackers to listen to traffic, intercept it, and spoof both ends of the data, constitute the MITM attack [48,49].
2. *Denial of Service (DoS)*: This type of attacks occurs also at the network layer by jamming the transmission of radio signals, using a fake node, affecting the transmission or routing of data between nodes [50,51].
3. *Eavesdropping/sniffing*: This type of passive attacks gives the intruder the ability to listen to the private communication over the communication link [52]. The intruder might be able to extract useful information such as usernames and passwords, node identification or node configuration, which could lead to other types of attacks, e.g., fake node, replay attack, etc.
4. *Routing attacks*: This type of attacks affects how the messages or data are routed. The intruder spoofs, redirects, misdirects or even drops packets at the network layer. The following specific attacks could be considered:
  (a) Black Hole: It can also be considered as a DoS attack, in which the intruder uses a fake node that welcomes all traffic by asserting that it has the shortest path. As a result, all traffic will be redirected to the fake node that has the ability to redirect them to a proxy server or even drop them [53].
  (b) Gray Hole: This type of attacks is similar to the black hole attack but instead of dropping all the packets, it only drops selected ones [54,55].
  (c) Worm Hole: In this type of attacks, the intruder creates a connection between two points in the network by either controlling at least two nodes of the network or adding new fake nodes to the network. After forming the link, the intruder collects data from one end and replays them to the other end [56,57].
  (d) Hello Flood: The aim of the attacker in this type of attacks is to consume the power of nodes in the system by broadcasting Hello request packets by a fake node to influence all the nodes in the system that they are in the same range, thus causing each one to send packets to its neighbor causing a huge traffic in the network [58,59,60]. (hello messages are defined in some routing protocols, so that nodes announce themselves to their neighbors.)
  (e) Sybil: In this attack, a fake node presents multiple identities, thus it can control a considerable part of the framework by being in different places within the network at the same time. When l many sybil nodes are within the same network, they will then send a large amount of information denying the normal nodes from using the network [61].

These potential attacks at the network layer (wired or wireless) lead to the definition of the following security requirements: hop-to-hop encryption, point-to-point authentication, key agreement and management, security routing and intrusion detection [62].

#### 3.2.3. Application Layer Security Issues and Requirements

The application layer is responsible for providing services. It hosts a set of protocols for message passing [19,63], e.g., Constrained Application Protocol (COAP), Message Queuing Telemetry Transport (MQTT), Extensible Messaging and Presence Protocol (XMPP), Advanced Message Queuing Protocol (AMQP), etc. This layer directly interacts with the user. Given that the “traditional” application-layer protocols do not perform well within IoT, and since the IoT does not have its own international standards, several security issues arise at the application layer [27].

1. *Data Accessibility and Authentication*: Each application might have many users [64]. Fake or illegal users could have a great impact on the availability of the whole system. Such great number of users means different permission and access control.
2. *Data privacy and identity*: The fact that IoT connects different devices from different manufacturers leads to the application of different authentication schemes. The integration of these schemes is a challenging issue to ensure data privacy and identity.
3. *Dealing with the Availability of Big data*: IoT connects a huge number of end devices, which leads to a huge amount of data to be managed. This causes an overhead on the application to analyze this data, which has a big impact on the availability of the service(s) provided by the application.

Regarding the security requirements for the application layer, authentication is required while protecting the privacy of users (respectively, data). In addition, there should be an information security management scheme that includes resource management and physical security information management. Table 1 gives a summary of the security requirements of the three-layers in the IoT architecture.

In Table 1, it is clear that authentication is a core security mechanism that should applied at different layers. An IoT use case might need an authentication between the end devices and an intermediate device (gateway). The gateway should authenticate itself while sending data to the cloud, and the application (mobile or web) should be authenticated to the cloud in order to collect data for analysis.

## 4. Taxonomy of IoT Authentication Schemes

**Figure 2:**
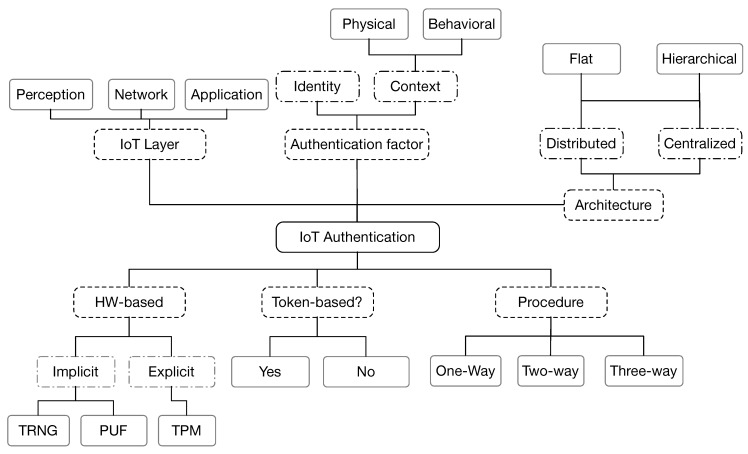
Taxonomy of IoT authentication schemes.

This section presents a taxonomy of IoT authentication schemes using various criteria selected based on the similarities and the main characteristics of these schemes [1,2]. As previously mentioned, the authentication can be applied at each of the three layers of the IoT architecture, which makes the diversity of the authentication techniques. These criteria are illustrated in Figure 2 and summarized as follows.

1. *Authentication factor*
  - Identity: An information presented by one party to another to authenticate itself. Identity-based authentication schemes can use one (or a combination) of hash, symmetric or asymmetric cryptographic algorithms.
  - Context: which can be:
    - Physical: Biometric information based on physical characteristics of an individual, e.g., fingerprints, hand geometry, retinal scans, etc.
    - Behavioral: Biometric based on behavioral characteristics of an individual, e.g., keystroke dynamics (pattern of rhythm and timing created when a person types), gait analysis (method used to assess the way we walk or run), voice ID (voice authentication that uses voice-print), etc.
2. *Use of tokens*
  - Token-based Authentication: Authenticates a user/device based on an identification token (piece of data) created by a server such as OAuth2 protocol [65,66] or open ID [67].
  - Non-Token based authentication: Involves the use of the credentials (username/password) every time there is a need to exchange data (e.g., TLS/DTLS [12,68,69]).
3. *Authentication procedure*
  - One-way authentication: In a scenario of two parties wishing to communicate with each other, only one party will authenticate itself to the other, while the other one remains unauthenticated.
  - Two-way authentication: It is also called mutual authentication, in which both entities authenticate each other.
  - Three-way authentication: Where a central authority authenticates the two parties and helps them to mutually authenticate themselves.
4. *Authentication architecture*
  - Distributed: Using a distributed straight authentication method between the communicating parties.
  - Centralized: Using a centralized server or a trusted third party to distribute and manage the credentials used for authentication.
  Whether centralized or distributed, the authentication scheme architecture can be:
  - Hierarchical: Utilizing a multi-level architecture to handle the authentication procedure.
  - Flat: No hierarchical architecture is used to deal with the authentication procedure.
5. *IoT layer*: Indicates the layer at which the authentication procedure is applied.
  - Perception layer: Responsible for collecting, processing, and digitizing information perceived data by the end nodes in IoT platform.
  - Network layer: Responsible for receiving the perceived data from perception layer and processing it.
  - Application layer: Responsible for receiving data from the network layer, and then providing services requested by users.
6. *Hardware-based*: The authentication process might require the use of physical characteristics of the hardware or the hardware itself.
  - Implicit hardware-based: Uses the physical characteristics of the hardware to enhance the authentication such as Physical Unclonable Function (PUF) or True Random Number Generator (TRNG).
  - Explicit hardware-based: Some authentication schemes are based on the use of a Trusted Platform Module (TPM), a chip (hardware) that stores and processes the keys used for hardware authentication.

## 5. Analysis of IoT Authentication Schemes

This section surveys the literature of the authentication schemes for IoT (including those defined for WSN). The analysis is based on the multi-criteria classification presented in Section 4.

The surveyed research works are organized based on the IoT application domains.

### 5.1. Smart Grids

Smart grid is taking the momentum over traditional power grids due to its efficiency and effectiveness, but security issues are still challenging in such field. In [70], the authors proposed an authentication scheme based on a Merkle-hash tree. Each home is equipped with a smart meter to collect the consumption of electricity for a time interval and sends the data via wireless communication to the Neighborhood Gateway (NG). The NG sends these data to the control center to collect the bill, which is sent back to the customer. The main contribution is the mutual authentication done between the smart meter and NG using a lightweight scheme that has an efficient computation and communication overhead. The authors of [71] also provided an effective and robust approach to authenticate aggregated power usage data in Neighborhood Area Network (NAN) with a fault-tolerance architecture, which is based on digital signature. In [72], the authors proposed a protocol to authenticate Home Area Network (HAN) smart meters with the smart grid utility network, and provided a new approach for key management.

While developing a smart grid, two main features should be taken into consideration: the data sent to the control unit or gateway should be sent from a valid smart meter, and there should not be a way of bring out the style of the customer by analyzing his consumption of electricity, thus breaking his privacy. In [73], the authors took the above features into consideration and developed an authentication scheme (called PASS) for smart grids based on Hash-based Message Authentication Code (HMAC). Such approach also ensures the privacy of the customer.

To achieve a lightweight message authentication scheme for smart grid, the authors of [74] built an approach that allows smart meters to mutually authenticate to other system components and achieve message authentication. The authentication is done using a lightweight Diffie–Hellman and the data integrity is achieved using HMAC.

In [75], the authors proposed an authentication protocol for smart grids called Smart Grid Mutual Authentication (SGMA) and another scheme called Smart Grid Key Management (SGKM) for key management. The scheme benefits from the traditional protocols and enhances them to achieve mutual authentication and key management. It uses the Secure Remote Password (SRP) [76] that depends on the password entered by the requester to generate a verification ID for further communications, but the enhanced version has less overhead with respect to the exchanged handshake messages and number of packets. Public Key Infrastructure (PKI) is used for key management but due to its overhead regarding the key regeneration, an enhanced version of ID-based Cryptography (EIBC) is used with PKI by replacing the public key with the identity of the requester.

In [77], the authors proposed a lightweight authentication protocol for smart grids. It consists of three tiers by using three different protocols for different purposes: Diffie–Hellman is used as key agreement protocol, with the use of RSA and AES for achieving the confidentiality, and HMAC for maintaining message integrity.

To address some performance and security challenges such as the storage cost and the key management, the authors of [78] discussed one-time signature to be used for multicast authentication in smart grids. The authors of [79] proposed a Privacy-preserving Recording and Gateway-assisted Authentication (PRGA) protocol for a hierarchical smart grid network with a gateway-based authentication. The scheme is based on HMAC and homomorphic encryption to authenticate and aggregate the messages sent by smart meters before sending them to the control center. This reduces the amount of exchanged data. The main advantage of such scheme is keeping the identity of the smart meter hidden to the gateway and the control center until the time of generating the bills, thus ensuring privacy.

Due to the limitation of one-time signature with respect to the size and the storage of the signature, the authors of [80] proposed a one-time signature-based multicast authentication in smart grid. Their scheme deployed a new one-time signature based on a nonlinear integer programming that reduces the computation cost.

### 5.2. RFID and NFC-Based Applications

Radio Frequency Identification (RFID) is a wireless technology that consists of tags that can be attached to any physical object or even humans; its main purpose is the identification or detection of the tagged objects. RFID can be deployed in various fields, e.g., supply chain, health care, climate sensing, etc.

In [81], the authors suggested a lightweight authentication protocol for RFID tags based on PUF. The protocol consists of three transactions: tag recognition, verification, and update. In the first transaction, the tag reader recognizes the tag. The second transaction is the verification, where the reader and the tag mutually verify the authenticity of each other. In the last transaction (Update), one should keep up the most recent used key for the next verification.

To protect the supply chain of connected devices, the authors of [82] enabled authentication and perceptibility of the IoT devices, through an RFID-based solution. The authentication process consists of two steps: checking the connectivity between the tag and the IoT device and then approving the perceptibility of the tag.

In an IoT-RFID based system, the RFID reader is connected to the Internet to form an IoT end device. On the other side, it is connected to the tagged items via RFID communication protocols. The tagged item is portable and moves from a reader to another, thus there is a need for verifying the identity of each other via authentication. Due to the absence of cryptographic features in RFID, the system is vulnerable to security threats such as impersonation or cloning attacks. In [83], the authors presented an authentication protocol to be used in IoT-RFID use case with the use of lightweight encryption algorithm.

To resist against cloning attacks to the RFID tag, the authors of [84] proposed an offline authentication for RFID-tags based on PUF. It combined both identification and digital signature security protocols. In the authentication, the tag generates a secret key by challenging the PUF and collecting the response. Such response with the helper data will create a certificate that will be stored inside the ROM of the tag. Next, the verifier authenticates the tag by checking the validity of the certificate. To provide anonymous authentication for RFID systems, the authors of [85] presented a PUF-based authentication scheme for classic RFID tags. Then, they provided an enhanced scheme for noisy PUF environment. The main drawback of such scheme is not taking into consideration re-feeding the server with new Challenge–Response Pair (CRP) when the existing pool becomes empty.

The authors of [86] proposed a two-factor authentication scheme based on smartphone with Near Field Communication (NFC) feature as first factor and fingerprint of the user as the second factor. Both factors are used to authenticate user on smart library system. The library database then checks if the personal data embedded in the NFC tag and the fingerprint match then the user is authorized to access the internal library network to query for books and providing the user with the rack position (location) in which the book is located.

The authors of [87] proposed a mutual authentication scheme for IoT RFID-based applications in fifth-generation mobile networks (5G) by providing the reader with a cache, to store the keys (used for authentication) for the recently visited tags, to speed up the authentication, reduce the computation cost and increase the security in storage.

The authors of [88] proposed a mutual authentication scheme for IoT NFC-based applications in 5G. By using only (lightweight) Shift and XOR operations to suit the performance and storage features of NFC tags, they provided the anonymity of the tags using pseudonym instead of the real identity.

### 5.3. Vehicular Networks

Cars nowadays are connected to the Internet or the Internet of Things to form what is called vehicular networks or Internet of Vehicles. Such connectivity is used to provide different services: providing traffic information for users, sharing riding services, charging electrical cars, etc. The field of Electrical Vehicles (EV) is becoming a trend and vehicle authentication is a challenging topic in EV systems. The authors of [89] proposed a two-factor authentication scheme for EV, although it can be deployed in different fields. The scheme combines unique contextual feature. The vehicle is connected to the server via a wireless connectivity and to the charger via a charging cable, so it depends to the physical connectivity to verify the identity.

The authors of [90] proposed an authentication protocol referred to as distributed aggregate privacy-preserving authentication (DAPPA) that can be used to authenticate a vehicle system with the use of multiple trusted authority one-time identity-based aggregate signature technique. A vehicle can verify many messages at the same time, and their signatures can be aggregated into a one message, which decreases the storage space needed by a vehicle or a data collector. In addition, in [91], the authors proposed a protocol to authenticate communication in secure vehicular ad-hoc networks (VANETs) using identity-based aggregate signatures.

In [92], the authors provided a broadcast authentication scheme called Prediction-Based Authentication (PBA) that defends against DoS attacks and resists packet loss. The protocol is based on Merkle hash tree construction for instant verification of urgent messages, and self-generated Message Authentication Code (MAC) storage instead of storing all the receiving signatures. As an enhancement to the scheme in [92], the authors of [93] provided a Prediction-Based Authentication (PBA) as a broadcast authentication scheme in VANETs. The proposed scheme utilizes both the Elliptic Curve Digital Signature Algorithm (ECDSA) signatures and Time Efficient Stream Loss Tolerant Authentication (TESLA) to authenticate messages between vehicles which proved to be an efficient, effective, and authentication scheme.

In [94], an enhancement to Dual Authentication and Key Management Techniques in VANETs is proposed, by providing an authentication phase the first time the vehicle enters the network and a re-authentication phase when the vehicle moves from one coverage area to another without the need to re-iterating the entire verification process.

The authors of [95] provided a mutual authentication between vehicles using a Challenge Handshake Authentication Protocol (CHAP) that achieves both authentication and authorization, thus allowing vehicle-to-vehicle (V2V) charging using a converter-cable assembly.

The authors of [96] proposed an authentication protocol for securing VANETs called (ESPA). The protocol uses asymmetric cryptography (PKI) and the symmetric HMAC for vehicle-to-infrastructure (V2I) and vehicle-to-vehicle (V2V) communications. In [97], the authors proposed a new model for authentication in VANETs based on group authentication. Vehicles are grouped and a session key is generated to be used for V2V communication.

The authors of [98,99] proposed a threshold authentication protocol to support secure and privacy-preserving communications in VANETs. The protocol is described by the use of a group signature scheme for achieving threshold authentication, efficient cancellation, anonymity, and traceability during the communication of vehicles. In [100], the authors used PKI to certify both the public key and a list of fixed unchangeable attributes of the vehicle. In [101], the authors proposed a scheme to prove the identity of one vehicle driver in to another during communication. Elliptic-curve cryptography (ECC) and steganography techniques are used for the authentication and privacy preservation of users.

In most VANETs, there was a problem of connecting it to IP networks using road-side access routers (ARs) due to obstacles and mobility of the vehicle. Hence, the authors of [102] studied the ability to provide IP connectivity to the vehicles in VANETs using a multi-hop scheme. The location and road traffic information are stored in a location server used for roaming purposes. The approach provides a mutual authentication between the roaming vehicle and ARs.

### 5.4. Smart Homes

Smart Homes are automated homes, where users will have the ability to control, monitor and access remotely ( using mobile phone (mobile application) or personal computer (web application)) climate systems (heating and air conditioning), appliances, lighting, TV, audio and video systems, etc. The authors of [66] developed a security scheme that can be deployed in smart homes. Such approach has the ability to overcome some security threats such as impersonation and replay attack [65], but it is still vulnerable to eavesdropping. The approach is based on OAuth 2.0 protocol. In [103], the authors introduced a new authentication scheme to authenticate the end devices deployed in smart homes, which is based on the combination of PUF and Physical Key Generation (PKG). The PUF provides the security of the system by generating a secure key based on the physical parameters of the end device ( the design of PUF depends on common circuit fabrication features that give it the ability to create unique secret key). Machine-to-Machine (M2M) communication is taking a lead in the IoT development, but it also has security challenges. In [104], the authors developed a scheme allowing a smart home network remote user to communicate with end devices. The scheme allows the mutual authentication between the communicating parties, besides establishing a secure connection to establish the confidentiality of data. The authors of [105] proposed a mutual authentication protocol for IoT end-devices (smart home use case) based on PUF. They introduced the concept of Object Life cycle (OLC) to describe the roadmap of the end-device from manufacture till the deployment in the IoT system and describing the security challenges during this roadmap.

The authors of [106] developed a PUF-based authentication scheme for IoT devices to provide mutual authentication between the end device and the gateway by using the CRP data stored inside the gateway. It also provides a way for user (smart phone or wearable device) to authenticate itself with the gateway in order to have the ability to communicate with the end devices using session key generated between them. Timestamp data are used by the user to ensure security against replay attacks.

The authors of [107] proposed a mutual authentication for IoT systems. The scheme is based on the lightweight features of Constrained Application Protocol (CoAP) as an application layer protocol for the communication between client and the server. The secure communication channel is provided by the advantage of Advanced Encryption Standard (AES) cipher. Both the client and the server challenge each other for mutual authentication by encrypting a payload from the message of size 256 bits, and then exchange payloads for verification. The authentication is done during the request-response interaction without the use of an extra layer (DTLS) which increases the communication and computation cost.

### 5.5. Wireless Sensor Networks

Wireless Sensor Networks (WSN) is the ability to add the connectivity and sensing features to billions of sensors embedded in various fields (appliances at homes, vehicles, grids, etc.).

The authors of [108] proposed an authentication protocol at the media access control sub-layer called Optimization of Communication for Ad-hoc Reliable Industrial networks (OCARI), in which they used a one-time shared session key. This technique is suitable for resource-constrained devices. Blom key predistribution scheme [109] and the polynomial schema [110] are considered as appropriate to be used as key management protocols for some IoT use cases.

The authors of [111] proposed an improved user authentication and key management protocol for WSNs using Bio-hashing [112]. The use of the BAN-logic ( logic of belief and action that ensures one part of communication believes that the key in authentication is good) ensures mutual authentication.

The authors of [113,114] proposed a mutual authentication which consists of two stages: in the enrollment stage, every node should be identified within the system, and in the authentication stage, a number of handshake messages are exchanged between the end device and the server, which result is a session key to be used for upcoming communication.

The authors of [115] developed a group authentication technique in wireless networks for achieving both mutual authentication and privacy. An end node will have the ability to move between access points without the need for re-authentication every time. All the roaming group members information are transmitted to the Base Station (BS) the first time a Mobile Station (MS) is authenticated. Then, the MS sends the information to the group manager, which aggregates all the information and sends it back to the BS. The BS sends it to the access service network for verifying the authentication. After analyzing some existing chaotic-maps based protocols, the authors of [116] provided a chaotic maps-based mutual authentication in WSN.

In [117], the authors proposed a lightweight authentication scheme with anonymous features by providing hop-by-hop authentication and un-traceability. The main contribution is the achievement of user privacy in WSN. In [118], the authors provided an authentication protocol for WSN, which can guarantee different security features such as the privacy of the user, un-traceability, backward secrecy, and strong forward secrecy. It is also resilient to node capture and key compromise impersonation attack.

To ensure mutual authentication between the user, the end node, and the gateway node (GWN) in a WSN, the authors of [119] developed an authentication scheme based on lightweight Hash and XOR operations that gives the remote user the ability to connect to the end nodes in WSN systems without the need to be connected at the beginning to the gateway.

Due to the resource-constrained features of the sensor nodes in WSN systems, there is a need for lightweight encryption protocols. Thus, the authors of [120] proposed a secure authentication protocol for WSN based on Elliptic Curve Cryptography (ECC) which is considered lightweight compared to the traditional RSA cipher. Besides, the authors applied Attribute-Based Access Control (ABAC) authorization mechanism for access control, which is considered scalable.

In [121], the authors proposed an authentication scheme to be deployed in WSNs. The scheme consists of two main steps: the initialization step in which the user is given a public/private key using a lightweight ECC-based protocol. The second step is the authentication step in which two nodes will use the public/private pair to mutually authenticate each other.

The authors of [122] provided a protocol called E-SAP for Efficient-Strong Authentication Protocol for wireless health care applications. This protocol consists of a number of features: a two-factor authentication (smart card and password), mutual authentication between sensors, symmetric cryptography to ensure message confidentiality and the ability to change passwords.

In [123], the authors provided a two-factor-based user authentication scheme for WSN using both passwords and smart cards. The approach is resistant to stolen-verifier and impersonation attacks, but it does not take into consideration node impersonation attack.

The authors of [124] proposed a new anonymous authentication scheme for IoT based on the lightweight ECC. The protocol is composed of two main steps: the registration step and the authentication step. A comparison is held between the ECC and RSA with respect to the time spent for encryption and decryption using different sizes of key.

The authors of [125] presented a secured way for the authentication of devices in Internet of Medical Things (IMT) using the physical characteristics of people for identification. The authors provided a survey of all the biometric techniques used in the literature especially in the smart health environments with an analysis regarding their compatibility with the IMT, a security analysis is also provided. Finally, the authors provided requirements and open issues for researchers and developers while using biometric properties for authentication.

The authors of [126] proposed a novel mutual authentication and key management for WSN systems based on biometric and symmetric crypto-system. They compared their scheme with the schemes provided in the literature regarding computation and communication cost and security threats using both BAN-logic and AVISPA [127] tools.

### 5.6. Mobile Network and Applications

To allow remote users to access Internet services any time, anywhere, the authors of [128] proposed a new scheme to provide a secure roaming for anonymous users benefiting from the group signature technique. They call it Conditional privacy-preserving authentication with access linkability (CPAL). In [129], the authors proposed two authentication schemes, the first one is based on pseudo-random authentication and the second is based on zero-knowledge authentication for providing authentication and location privacy-preserving scheme for LTE-A. The schemes enable all the entities in LTE-A networks to mutually authenticate each other and update their location without involving the subscriber server. The authors of [130] provided also a group-based authentication scheme for LTE networks by developing a group temporary key. It is based on both Elliptic Curve Diffie–Hellman (ECDH) that provides forward and backward secrecy, while using asymmetric key protocol to provide user’s privacy. The authors of [131] proposed SEGR for the authentication of a group of devices using both 3GPP or WIMAX systems. It is based on certificate-less aggregate signature which was proposed to remove the complication of certificate management in public key cryptography.

Due to challenging issues in developing a user-friendly authentication scheme for smart phone environment where touch screen is the most user-friendly input peripherals, the authors of [132] provided an authentication process for Android smartphone devices using dual-factor authentication called (Duth). The protocol is made up of a registration step in which the place and time of user entering patterns to the touch screen are stored and then the stored data are used as dual factors for authentication. This approach can improve security without adding any extra hardware.

The authors of [133] proposed a new authentication scheme for mobile phone users based on behavioural pattern. They started by collecting the behavior of mobile phone user regarding the applications used in a specific time and the duration of usage, and then they change these data to a unique pattern to be used as an authentication between the user and the mobile phone.The proposed scheme will be used as complementary to the existing authentication schemes provided by mobile phones (pin code, fingerprint, gestures, etc.).

### 5.7. Generic IoT Applications

The authors of [134] provided a protocol for performing authentication between a user and a server, and not between end devices. It is a two-step verification to IoT devices. A password or a shared secret key is considered as the first authentication factor and the use of PUF as the second authentication factor.

Due to the large number of devices wishing to access the network which leads to an overload for the authenticating server and in order to achieve mutual authentication and secure key management for resource-constrained devices, the authors in [135] provided a group-based lightweight authentication and key agreement scheme called GLARM. GLARM is composed of two fundamental stages: an identification stage and, a group authentication and key-agreement stage based on a combination of message authentication code of a group of devices to be authenticated.

In [136], the authors proposed a lightweight device authentication protocol for IoT systems named speaker-to-microphone (S2M). This scheme achieves distance authentication between wireless IoT devices. Is is implemented in both mobile phones and PC to check its experimental results.

The author of [137] provided a new hardware-based approach, using a hardware fingerprint to authenticate IoT device with its Physical Unclonable Functions (PUF). The author presented the machine learning based attacks on PUF leading to the creation of a software model of the PUF. The author of [138] presented an implementation of PUF-based algorithms for IoT device. They used PUF-based elliptic curve for device enrollment, authentication, decryption, and digital signature. The author fingered the environment variations under which the IoT devices will be operating and also the effects of aging in the usage of PUF by using error correction codes. They handled the machine-learning attacks by combining the PUF with the use of ECC to encrypt the generated key. In [139], the authors proposed an authentication mechanism which applies a modeling for the PUF to avoid storing the whole Challenge–Response Pairs (CRPs) or the retrieval of some CRPs during authentication or verification phases, and the model will be hidden by applying a symmetric encryption algorithm (AES) to avoid modeling-based attacks.

The authors of [140] realized that a single-tier authentication is not adequate for services such as cloud computing. Thus, they proposed a multi-tier authentication scheme without the use of any extra software or hardware. In the first stage, user enters a simple username and password that is validated by the server on the cloud. In the second stage, user pursues a predetermined sequence on the virtual screen during the registration phase.

In [141], the authors provided an authentication scheme to be used in cloud computing use cases. They divided the devices into two categories: registered and unregistered devices, and handled the authentication using two different approaches. The registered devices are authenticated using an authentication server. Firstly, the device is registered in the server, then a session key is generated by the server and sent encrypted using Advanced Encryption Standard (AES), to the device to be used for further communications. On the other hand, a cloud-based Software-as-a-Service (SaaS) using modified Diffie–Hellman (DH) algorithm is used to authenticate unregistered devices for accessing cloud services.

Using password or smart cards as the only way of authentication for remote users is vulnerable to security attacks. Thus, the authors of [142] proposed a novel scheme by using a three-factor authentication. The fingerprint or iris-scan, the smart card, and a password to authenticate a user to remotely-based applications.

Although traditional Public Key Infrastructure is considered heavy with respect to the computation and communication cost, the authors of [143,144] presented a lightweight PKI to be implemented in IoT use cases. The authors of [145] presented an efficient transmission to ensure privacy of data sent. The proposed scheme used a customized data encapsulation to reduce both the computation and communication overhead.

The authors of [69] presented a two-way IoT authentication protocol. The authentication is handled using the Datagram Transport Layer Security (DTLS) protocol with the exchange of certificates based on RSA. This work is an enhancement of the work reported in [68], a two-way IoT authentication schemes based on RSA with the use of Trusted Platform Module (TPM).

In [146], the authors proposed an authentication protocol based on PUFs that uses the zero-knowledge proof of knowledge (ZKPK) of discrete logarithm. The protocol requires a user to input a password to the device each time it requires authentication. The limitation of this protocol is providing the authentication between the user end device and the server without also taking into consideration authentication between end devices. The use of ZKPK increased the complexity of this protocol.

After doing a security analysis for some of previous scheme for the authentication of devices in Low-earth-orbit satellite (LEOs) communication systems, the authors of [147] proposed an authentication and key management scheme based on Elliptic curve and symmetric cryptography. The proposed scheme provides a mutual authentication between the user and the Network Control Center (NCC), besides its resilience to stolen verifier, replay, DoS, password guessing, and impersonation attacks.

The authors of [148] presented a two-factor device authentication scheme. They used both the digital signature and a novel factor called the device capability. Device capability is similar to a functional operation solved by the device, which could be a mathematical challenge or even a cryptographic-based puzzle. Such scheme can be used to authenticate both the end-device and the server too. Using a secure TLS channel, the device sends a request for communication to the server, the server then sends a nonce encrypted with its private key and the timestamp to avoid replay attacks. The device then decrypts the signature, solves the nonce with functional operation, signs the result with its private key and sends it back to the server. The server then checks the valid signature and the result of the functional operation to authenticate the device.

The authors of [149] proposed a new authentication scheme for IoT system based on PUF. The scheme is based on generating a response of a challenge, then feeding the response to another PUF as a challenge. The two PUFs are connected using Linear Feedback Shift Register (LFSR). The main drawback of such design is its complexity, and the lack of security analysis. Machine learning attacks to create a model of PUF is not considered.

The authors of [150] proposed a new authentication scheme for IoT systems based on blockchain called Bubbles-of-Trust. The idea is to divide the devices into virtual zones called bubbles in which they identify and trust each other (concept of grouping). Then, the communication (transaction) between different devices is controlled and validated by the public blockchain implemented using Ethereum.

To provide authentication for IoT system integrated in cloud computing environment, the authors of [151] provided a lightweight two-factor authentication scheme based on one-way hashing and XOR operation. The authentication process is made up of three steps: registration, verification, and password renewal step. The computation cost of such scheme is considered and its efficiency is shown in resource-constrained environments.

To ensure the security of IoT systems, the authors of [152] took advantage of PKI by using X.509 digital certificates to ensure the authentication of devices within the system. Such certificates can be used for device identification and also the device integrity.

The authors of [153] proposed a model to addresses the IoT security issues. They used Object Naming service (ONS) to query the Domain Name Server (DNS) to search for information about a device using either its IP address or electronic product Codes (EPC). They ensured the anonymity of the requester by asking multiple ONSs to hide the identity and using multiple encryption layers using the encryption key of the router during transmission.

Table 2 summarizes the different surveyed IoT authentication schemes with a classification based on the taxonomy presented in Section 4 as well as a summary of their advantages and disadvantages.

The following abbreviations are used for the below criteria:

- IoT Layer: A, Application; N, Network; P, Perception.
- Procedure: 1, One-way; 2, Two-way; 3, Three-way.
- Architecture: C, Centralized; D, Distributed; F, Flat; H, Hierarchical.
- Hardware-Based: I, Implicit; E, Explicit.

## 6. Related Works

Several research works surveyed the IoT authentication solutions. The authors of [154] presented a survey of the authentication protocols deployed in Internet of Things (IoT) use cases by categorizing them into four categories: Internet of Sensors (IoS), Internet of Energy (IoE), Machine-to-Machine (M2M) communication, and Internet of Vehicles (IoV). A taxonomy and comparison of authentication protocols are provided in terms of: network model, goals, main processes, computation complexity, and communication overhead. In [155], the authors provided a classification and comparison of different authentication protocols for IoT. The authors created two different classifications: the first one based on whether the authentication technique is being distributed or centralized, flat or hierarchical, and the second one based on the characteristics of the authentication process. In [156], the authors provided an analytical survey of the existing literature. They outlined the potential issues and challenges of authentication in IoT for further research but without providing a comparison of the existing schemes. The authors of [157] provided different authentication methods used within the IoT context with emphasis on lightweight and mutual authentication methods but without classification or comparison. In [158], a review of the prominent and “recent” authentication techniques for IoT systems is provided with an indication of their limitations and future research trends, but without providing a classification. The authors of [159] provided a classification of selected research works addressing security concerns and authentication methods, in addition to their evaluation in terms of advantages and disadvantages. In [160], the authors mapped the current state of the art of the authentication in an IoT environment, featuring the difficulties and the primary methods utilized in authentication scheme but without performing any comparison. The authors of [161] provided a brief overview of authentication mechanisms with an evaluation of the proposed schemes in the literature. They also evaluated each method based on its resource consumption (e.g., energy, memory, computation and communication).

## 7. Conclusions

This paper presents a taxonomy and a literature review of authentication in the IoT context. The analysis of a large spectrum of authentication protocols/schemes leads to identify a number of requirements and open issues that should be taken into consideration by researchers and developers while developing new authentication schemes for IoT networks and applications.

1. In sensor-based applications, where sensors (constrained in terms of memory, processing power, battery, etc.) are the main end-devices, the proposed protocols must be lightweight, making a trade-off between power consumption and security.
2. The robustness of authentication protocols against potential attacks, e.g., sybil, node capture, replay, password guessing, message forgery, brute force, man-in-the-middle, DoS, collision, chosen-plaintext, etc. should be considered and analyzed. In particular, it is important to to consider Distributed Denial of Service attacks (second attack against IoT in 2017 as per [6]).
3. There is a need to consider location and identity privacy in certain IoT applications especially smart grids and VANETs.
4. The communication overhead of authentication protocols is a key factor, especially when dealing with power-limited devices; the number of messages exchanged between authentication parties should be kept as low as possible. In the same context, the size of the messages should be as small as possible due to the restricted bandwidth of the wireless communication protocols used.
5. Low computation cost should be considered while designing IoT authentication schemes especially for power-constrained and processing-limited IoT environment. This emphasizes the need to adopt lightweight cryptographic algorithms and protocols while designing authentication solutions.
6. IoT authentication scheme should be scalable in the sense that it should manage large number of nodes s well as have the ability to add new nodes without any further setup or configuration.
7. Authentication service should be ensured for the three layers of IoT architecture (application, network and perception layer).
8. Heterogeneity of devices in IoT networks must be taken into consideration while designing IoT authentication schemes.
9. Hardware security using “PUF” is the current trend due to its advantages over software security approaches. A combination between software solutions (lower cost) and hardware solutions (more secure) should be considered.

## Figures and Tables

**Table 1:** IoT Architecture and security requirements.

Layer	Security Requirements
**Perception**	Lightweight Encryption
	Authentication
	Key Agreement
	Data Confidentiality
**Network**	Communication Security
	Routing Security
	Authentication
	Key Management
	Intrusion Detection
**Application**	Authentication
	Privacy protection
	Information Security Management

**Table 2:** Analysis of IoT Authentication Schemes.

Ref#	IoT Layer	Identity Context Credentials	Token Based?	Proc.	Arch.	HW.	Strength(+)/Weakness(−)
[145]	A	Encryption	x	2	C/H	I	+Encapsulation is used to efficiently benefit from resources.
[69]	A + N	Encryption/RSA	x/DTLS	2	C/H	I	+Low overhead regarding computation and communication and high interoperability −Unreliability due to the use of UDP over DTLS leads to
[108]	P	Encryption/Symmetric asynchronous One Time Password (OTP)	x/TLS	2	C/H	I	+Resistance to some DoS and replay attacks. −Performance analysis is not considered.
[109]	N	Encryption/Symmetric	✓(NodeID, Indices of space, and Seed)	3	D/H	I	+Resistance to node capture attack −Energy cost is not efficient.
[110]	N	Encryption/Symmetric	✓(polynomialID)	3	D/H	I	+Resistance to node capture attack +Efficient with respect to communication overhead −No consideration to location privacy.
[143]	A + N	Encryption/Asymmetric	✓(Information)	2	D/H	I	+Resistance to Malicious entity by using PKI −Performance analysis is not considered
[144]	A + N	Encryption/Asymmetric	✓(FormId)	2	C/H	I	+Compatibility problems are solved −Performance analysis is not considered.
[153]	A + N + P	Encryption/Symmetric	✓(IP/EPC)	2	C/F	I	+Resilience to attacks, access control, client privacy and data confidentiality
[83]	N + P	Encryption/Symmetric using XOR	✓(SpecificId)	2	C/F	I	+Authentication of RFID tags with readers −No consideration of location privacy.
[119]	N + P	Encryption/Symmetric + Hash + Credit card as context	✓(Nonce)	2	C/H	E	+Resistance to Man-in-the-Middle, Impersonation, Replay, Privileged insider attacks, Stolen smart card, Smart card breach attacks, etc. −Communication cost is not efficient.
[120]	N + P	Encryption/Asymmetric using ECC	✓(Identity, Elliptic curve function, and parameters)	2	C/H	I	+Resistance to Eavesdropping, DoS, Node capture, Replay and MITM attack −Attribute-based access control should be discussed in detailed manner.
[113]	A + N + P	Encryption/Asymmetric using RSA and ECC	x/DTLS	2	C/F	I	+Performance measurement is considered. +Resistance to MITM attack. −DoS and Replay attacks are not considered.
[114]	A + N + P	Encryption/Asymmetric using ECC	✓(Identity)	2	D/F	I	+Resistance to malicious users and DoS attacks. −Not Efficient regarding the storage of the certificate. −Vulnerable to node capturing attack
[65,66]	A	N/A	✓(OAuth2.0)	1	C/H	I	+Resistance to Impersonation and Replay attacks −No performance analysis is done. provided
[141]	A	Encryption: registered user with AES and non-registered users using Diffie–Hellman	x (username + password)	1	C/F	I	+Two separate servers for storing cryptography and authentication data, +Resistance to brute force, timing, and MITM −No performance analysis is done.
[82]	P	Encryption/Symmetric using AES	✓	2	C/H	I	+Resistance to split attacks (i.e., swapping tags, separating tag from product, etc.) −No analysis is done for location privacy.
[162]	A	No Encryption/Hash	✓	1	D/F	I	+Resistance to MITM, replay, DoS, and Eavesdropping attacks. −Storage cost is not efficient
[140]	A	Context/multiple credentials using physical context	x (User name + password)	Multiple authentication	D/H	I	+Resistance to replay attack −DoS attack is not considered
[142]	A	No Encryption/Hash 256 bits + context bio-metric	✓(User Identity)	2	D/F	E	+Second-tier authentication is done at client side. +Resistance to attacks from inside the system. −No ability to change credentials in both the tiers.
[121]	A	Encryption/Asymmetric using ECC	✓(UserId)	2	D/F	I	+Resistance to MITM and DoS attacks. −User must be authenticated many times in Multi-server environment.
[79]	A	Encryption/RSA + Hash SHA or MD5	x	2	C/H	I	+Filtering of the messages at gateway +Privacy preservation is considered +Resistance to Brute force, DoS, and Replay attacks −DDoS is not considered.
[77]	A + N	Encryption/RSA + AES	x	2	D/F	I	+Resistance to Modification, Replay, and Message analysis attacks −No analysis done for location privacy
[111]	A + N	Encryption/Symmetric + Hashing	✓ + x	2	C/F	I	+Resistance to Replay, Impersonation, Spoofing, and Gateway attacks. −Blackhole and Wormhole attacks are not studied
[135]	A + N + P	Encryption/Symmetric	✓(Identity)	2	C/F	I	+Resistance to MITM and DoS attack −Identity and Location privacy is not considered
[130]	A + N + P	Encryption/Asymmetric using ECC	✓(Identity)	3	C/F	I	+Resistance to redirection, malicious, Dos, and MITM attacks. −Location and Identity privacy is not analyzed and can not authenticate group of machines at the same time
[129]	N + P	Encryption/Asymmetric	✓(Identity)	2	C/F	I	+Location privacy is analyzed +Low communication and computational cost.
[131]	N + P	Encryption/Asymmetric using ECC + Hashing	✓(Identity)	2	C/F	I	+Group of end devices is authenticated at the same time −Location and Identity privacy are not analyzed and resistance to attacks are not studied
[136]	N + P	N/A	✓(audio samples as an identity)	2	D/H	I	+Low error rate and resistance to audio replay, changing distance, and same type device attacks. −Location privacy is not analyzed
[115]	N + P	Encryption/Symmetric + Hashing + Asymmetric using ECC	✓(Elliptic curve function)	2	C/F	I	+Privacy preservation is considered −Threat and attacks are not considered
[104]	A + N + P	Encryption/Symmetric(AES) + Hashing	✓(user Identity)	2	C/F	I	+Resistance to guessing, impersonation, and replay attacks −Privacy preservation is not considered
[128]	N + P	Encryption/Symmetric + Asymmetric using Hybrid Linear Combination Encryption (HLCE)	✓(user Identity)	2	C/F	I	+Resistance to DoS and impersonation, considers data integrity and ensures user privacy −Location privacy is not analyzed.
[132]	P	N/A	✓(writing process)	2	C/H	I	+Improve security without adding any extra hardware −No analysis for threats and attacks is done.
[102]	A + N + P	Encryption/Symmetric	✓(user Identity)	2	D/H	I	+Less location update.+Uses asymmetric links in VANET. +Resistance to MITM, replay, and DoS attacks −Privacy is not analyzed
[98,99]	N + P	Encryption/Symmetric + Asymmetric + Hashing	x	3	D/H	I	+Computation cost is efficient. +Resistance to the replay attack −No analysis is done to the communication overhead
[92]	N + P	Encryption/Symmetric+ Hashing	x	2	C/F	I	+Resistance to DoS attacks and to packet loss +Computation and storage overhead are low −Privacy preservation is not considered
[93]	N + P	Encryption/Symmetric+ Hashing	x	2	C/F	I	+Resistance to DoS attacks and to packet loss +Computation and storage overhead are low. −Privacy preservation is not considered
[101]	N + P	Encryption/Asymmetric using ECC	✓(cover image)	2	C/H	I	+Error rate is considered and analyzed −The size of the cover image should be greater than or equal the size of the text message. Otherwise, the text message will be truncated −Attack analysis is not considered.
[94]	N + P	Encryption/Symmetric + Asymmetric + Hashing	✓(Spanish eID cards)	2	C/H	E	+Resistance to replay, DDoS attacks and improve confidentiality and non-repudiation. +Computation and communication overhead is low +Location privacy is considered −Identity privacy is not considered
[95]	N + P	Encryption/Symmetric(AES) + Asymmetric (DH) + Hashing	✓(transaction ID)	2	C/H	I	+No rely on third parties for shared key management, instead a DH based key exchange mechanism is used. +Resistance to impersonation and MITM attacks. −Identity privacy is not considered
[96]	N + P	Encryption/Symmetric + Asymmetric + Hashing	✓(pseudo-identity (PsID))	2	C/H	I	+Provides a node, message authentication, and privacy protection. +Computation and communication overhead is studied. −Identity privacy is not considered
[97]	N + P	Encryption/Symmetric(AES) + Asymmetric(ECDSA) + Hashing	✓(pseudo-identity (PsID))	2	C/F	I	+Security and privacy analysis is considered +Computation and communication overhead is considered −Identity privacy is not considered
[90]	N + P	Encryption/Asymmetric + Hashing	✓(pseudo-identity (PsID))	2	C/F	I	+Resistance to DoS, Replay, Sybil, and False message attacks. +Consider privacy preservation. −Non-repudiation attack is not considered
[91]	N + P	Encryption/symmetric + Hashing	✓(identity)	2	C/H	I	+Resistance to Movement tracking, Replay, and Message modification attacks. −Location privacy is not considered
[100]	N + P	Encryption/Symmetric + Asymmetric + Hashing	No/TLS	2	C/F	I	+Resistance to Impersonation and MITM attacks. −Privacy is not considered
[89]	N + P	Encryption/Symmetric + Asymmetric + Hashing	x	2	C/F	I	+Resistance to Substitution attack (a kind of MITM attack). −No evaluation for message delay and verification delay
[80]	A + N + P	Encryption/Asymmetric + Hashing	x	2	C/F	I	+Efficient use of one-way hash +Resistance to message forging attacks+Reduce the storage −Increase the computation overhead although it is balanced between sender and receiver −Privacy is not considered. −Integrity and Confidentiality are not considered.
[78]	N + P	Encryption/Asymmetric + Hashing	x	2	C/F	I	−Increase the computation overhead although it is balanced between sender and receiver −Storage cost is high −The distribution of the public key via secure channel is done before the complete consumption of the hash chains.
[70]	N + P	Encryption/Symmetric + Asymmetric + Hashing	x	2	C/F	I	+Resistance to Message modification attacks, Replay, Message analysis, and message injection attacks. +Efficient with respect to computation and communication overhead. −Most of the routing attacks are not considered.
[71]	N + P	Encryption/Asymmetric + Hashing	x	2	C/F	I	+Computation and communication cost is efficient +Fault tolerance Architecture. −Attacks and threats analysis is not considered.
[72]	N + P	Encryption/Asymmetric + Hashing	✓(Node ID)	3	C/F	I	+Resistance to DoS, MITM, Brute force and Replay attacks +Secure key management. +Computation and communication cost is efficient. −Confidentiality and Integrity are not considered
[73]	N + P	Encryption/Asymmetric + Hashing	✓(Identity)	2	C/F	I	+Efficient with respect to the success rate+Identity privacy is considered+Message overhead is low −Routing attacks are not considered −Storage cost is not considered
[74]	N + P	Encryption/symmetric + Asymmetric + Hashing	x	2	C/F	I	+Resistance to Replay, collision, and chosen-plain-text attacks. −Location and Identity privacy is not considered
[75]	N + P	Encryption/Asymmetric + Hashing	✓(Entity Id and Serial number)	2	C/F	I	+Resistance to Impersonation, Brute force, DoS, MITM, and Replay attacks +Reducing management cost −Storage cost is not considered
[116]	N + P	Encryption/Asymmetric(chaotic map) + Hashing	✓(Identity id and password)	2	C/H	I	+User is anonymous +Identity privacy is considered +Secure session key +Computation, communication and storage costs are considered −Data integrity is not considered
[117]	A + N + P	Encryption/symmetric + Asymmetric + Hashing	✓(Identity)	2	D/H	I	+Resistance to Password guessing, Impersonation, Forgery, and Known session-key attacks +Computation cost is considered −Location privacy and communication cost are not considered
[123]	A + N + P	Encryption/symmetric + Hashing	✓(Identity id and password)	2	C/F	E	+Resistance to Stolen-Verifier, Password guessing, and Impersonation attacks −Sensor node impersonation attack is not considered −Unsafe against user who have privilege within the system attack. −No way of changing the password.
[122]	A + N + P	Encryption/symmetric + Hashing	✓((smart card and password)	2	C/F	E	+Resistance to Replay, Masquerading user, Masquerading gateway, Gateway secret guessing, Password guessing, and Stolen-Verifier attacks +Computation and communication cost is analyzed −Unsafe against privileged insider and off-line password guessing attacks.
[118]	A + N + P	Encryption/symmetric + Hashing	✓(Identity Id)	2	C/F	I	+Resistance to node capture and key compromise impersonation attack. +Security is achieved even if the smart card is stolen or lost. −Message delay and verification delay is not considered.
[137]	P	N/A	x	2	C/F	I	+Provided a discussion of machine learning attacks and how to deal with it. −Lack of dealing with the variation of environmental conditions
[103]	P	Encryption/Asymmetric(RSA or ECC) + Hashing	x	2	C/F	I	+Resistance to MITM and compromising a device attacks. −Lack of dealing with the variation of environmental conditions
[138]	P	Encryption/symmetric + Asymmetric + Hashing	x	2	C/F	I	+Resistance to machine-learning attacks +Deal with environmental variations by using error correction codes
[146]	P	Hashing	✓(User password)	2	C/F	I	+Resistance to device cloning or copying −An attacker could fake the user, then asking him for his password −Authentication is done between a server and end device only.
[134]	P	Encryption/symmetric + Hashing	✓(One-time alias, identity and nonce)	2	C/F	I	+Computation cost is low +Impersonation and Physical attacks is taken into consideration. − No consideration for machine-learning attacks. −No consideration for environmental variations
[139]	P	Encryption/symmetric + Hashing	x	2	C/F	I	+Protect against physical and side channel attacks +Avoid modeling attacks by hiding the CRP data −No consideration for environmental variations
[105]	P	Encryption/symmetric + Asymmetric + Hashing	✓(Dynamic Identity)	2	C/H	I	+Protect against Impersonation, Physical, and Reply attacks. −No consideration for machine-learning attacks. −No consideration for environmental variations
[84]	P	Encryption/symmetric + Asymmetric + Hashing	✓(Identity)	2	C/F	E	+Resistance to physical cloning attack. +Resistance to cloning attack based on attacking the communication protocol between the reader and the tag. −No consideration for environmental variations
[85]	P	Encryption/symmetric + Hashing	✓(Identity)	2	C/H	I	+Deal with environmental variations(noisy system) +Resistance to Physical attacks, DoS attacks, and Forward secrecy. +Efficient in terms of computation and communication cost+Security analysis is done compared to other schemes +Authentication scheme is evaluated using AVISPA tool. −No consideration for machine-learning attacks
[81]	P	Encryption/symmetric + Hashing	✓(nonce)	2	C/F	I	+Resistance to cloning, replay, back-tracking, and clone attacks. +Efficient in terms of computation −No consideration for environmental variations
[68]	A + N + P	Encryption/symmetric + Asymmetric + Hashing	✓(Identity)	2	C/F	E	+Resistance to DoS −Large packet header −Large key size of RSA.
[124]	P	Encryption/symmetric + Asymmetric + Hashing	✓(Identity + smart card + password)	2	C/H	E	+Compared with RSA regarding the encryption and decryption time −No security analysis is done
[106]	P	Encryption/symmetric + Hashing	✓(smart device ID)	2	C/H	I	+Efficient in terms of computation and communication +Resistance to Replay attacks −No consideration for machine-learning attacks −No consideration for environmental variations
[147]	A + N + P	Encryption/Asymmetric + symmetric + Hashing	✓(smart card + ID)	2	C/H	E	+Computation and communication +Resistance to replay, impersonation, stolen verifier, DoS, and offline guessing attacks. +Privacy preserving is considered. −Not efficient in terms of computation and communication.
[125]	A + N + P	Biometric	✓(physical properties)	1	C/H	E	+Ease to be used in smart health environments+No ability to be stolen, borrowed, and forgotten. +Resistance to clone and fork attacks. −The uniqueness of behavioral biometric properties is low and its computation cost is high.
[126]	A + N + P	Encryption/+ symmetric + Hashing + Biometric	✓(physical properties, smart card, ID and password)	2	C/H	E	+Uses three-factor authentication and privacy preserving is considered. +Resistance to replay, MITM, active, passive, forgery, user traceability +Resistance to clone and fork attacks. +The ability to update the biometric or password. +Scalability is high −No consideration for DoS and DDoS attacks.
[148]	P	Encryption/Asymmetric	✓(Nonce)	2	C/H	I	+Uses two-factor authentication and +Resistance to replay attack −No security analysis is done −No comparison is done with existing authentication schemes.
[149]	P	Encryption/+symmetric	✓(physical properties)	2	C/H	I	+New architecture using PUF +The ability to update the biometric or password. −No consideration for machine-learning attacks −Complex architecture with respect to resource-constrained devices in IoT
[150]	A + N + P	Encryption/+Assymmetric + Hashing	✓(ID, token)	2	C/H	I	+New architecture of using Blockchain in IoT. +Security analysis is done. −The consensus protocol takes about 14 seconds to validate a transaction which considered long period for real time applications −Using a public blockchain requires a fees to be paid for each transaction, which considered inefficient.
[107]	A + N + P	Encryption/+symmetric	✓(ID, token)	2	C/H	I	+Resistance to replay, DoS, eavesdropping, and resource exhaustion attacks. +Computation and communication cost is low. −Preshared key should be deployed at the provisioning phase −Privacy preservation is not considered.
[152]	A + N + P	Encryption/+ Assymmetric + symmetric + Hashing	✓(ID)	2	D/H	I	+Use of Azure IoT hub cloud infrastructure. +The accuracy of generating X.509 digital certificates for device authentication is increased from 50.9% into 84.7%. −Security analysis is not considered. −No consideration for the scalability requirements of IoT systems while using PKI.
[151]	A + N + P	Encryption/+ symmetric + Hashing	✓(ID, pseudo-identity pair)	2	D/H	I	+Computation cost is low at the device side. +Resistance to offline guessing, User tracking, forgery, and insider attacks. −Communication cost is high. −No consideration for Dos and DDoS attacks. −Computation cost at cloud is high.
[86]	P	physical fingerprint as context	✓(ID)	1	C/F	E	+Two-factors are used for authentication +Quick search of specific book and finding the location by attaching an NFC tag on each book. −Data collected are stored as plain text in the mobile phone. −Computation and communication cost is considered. −No security analysis is done.
[133]	P	Behavior as context	✓(ID)	1	C/H	I	+Modeling subset of the data to test the results −Restriction of the study to android platform mobile phones only −Data collected are stored as plain text in the mobile phone −No consideration for attacks. −Computation cost is high. −Achievement of positive identification of the owner is 70 out of 100.
[87]	P	Hashing + Xor	✓(ID)	2	C/H	I	+Novel scheme and benefiting from cache concept. +Resistance to forward, replay, eavesdropping, spoofing, tracking, and DoS attacks. +Computation and storage cost is efficient with respect to other schemes in literature. −Security of data storage in IoT systems is not considered.
[88]	P	Shifting + Xor	✓(ID, pseudonym (IDS))	2	C/H	I	+Novel scheme and benefiting from cache concept. +Resistance to forward, replay, eavesdropping, tracking, de-synchronization, and DoS attacks. +Computation and communication cost is efficient with respect to other schemes in literature. −Storage overhead of the database is high.
